# Threshold-induced correlations in the Random Field Ising Model

**DOI:** 10.1038/s41598-018-20759-6

**Published:** 2018-02-07

**Authors:** Sanja Janićević, Dragutin Jovković, Lasse Laurson, Djordje Spasojević

**Affiliations:** 1 Faculty of Physics, University of Belgrade, POB 368, 11001 Belgrade, Serbia; 2 COMP Centre of Excellence, Department of Applied Physics, Aalto University, P.O. Box 11100, 00076 Aalto, Espoo Finland; 3 Helsinki Institute of Physics, Department of Applied Physics, Aalto University, P.O. Box 11100, 00076 Aalto, Espoo Finland

## Abstract

We present a numerical study of the correlations in the occurrence times of consecutive crackling noise events in the nonequilibrium zero-temperature Random Field Ising model in three dimensions. The critical behavior of the system is portrayed by the intermittent bursts of activity known as avalanches with scale-invariant properties which are power-law distributed. Our findings, based on the scaling analysis and collapse of data collected in extensive simulations show that the observed correlations emerge upon applying a finite threshold to the pertaining signals when defining events of interest. Such events are called subavalanches and are obtained by separation of original avalanches in the thresholding process. The correlations are evidenced by power law distributed waiting times and are present in the system even when the original avalanche triggerings are described by a random uncorrelated process.

## Introduction

The Random Field Ising model (RFIM)^1,2^ has a great significance for studies of the effect of disorder in the ferromagnetic hysteresis^3^ and the Barkhausen effect^4–7^. In athermal (*T* = 0) and nonequilibrium case, the model has a disorder induced transition^8–10^ in dimensions 2 ≤ *d* < 6, while for dimensions *d* ≥ 6 it is described in terms of the mean field theory^11–14^. In this model, when the external magnetic field slowly changes, the system relaxes in spin-flipping avalanches, causing abrupt and jerky jumps of magnetization. This type of response to the external perturbation by means of a characteristic intermittent avalanche-like relaxation is immanent in many different physical systems exhibiting crackling noise^15^. Examples of such systems stem from earthquakes^16–19^ to neuronal networks^20^, from compression of wood samples^21^ and porous materials^22–24^ to deformation and fracture of stressed materials^25–27^, to name just a few.

One very important common feature of these systems is that such avalanche-like signals appear to exhibit complex temporal correlations. In this paper, we tackle this issue by investigating the distributions of waiting times between the consequtive avalanches in the RFIM signals at *T* = 0. We do this by analysing the data from numerical simulations and show how the appearance of the correlations can be attributed to the thresholding process we used to define the avalanches from the signal^28–30^. In the absence of correlations, the waiting times should follow the exponential distributions indicating the randomness of the avalanche triggering process. However, if the correlations are present, then one might expect a different functional form that the waiting time distributions should obey. We study this by applying the finite detection threshold on a generated signal, thus breaking the original avalanche sequence into smaller portions or subavalanches, and consequently implicitly imposing the correlations on a given signal. Similarly to the recently reported case of the other crackling noise system of crack line propagation^30^, we also find that the waiting time distribution, upon a process of thresholding, becomes of a power law type, indicating the onset of the apparent correlations in the system.

## Results

Thresholding is a procedure applied to data either intentionally, or effectively due to the device limitations in experimental measurements where the thresholding cannot be avoided^31^. So, let the response signal of the system under study be *V*(*t*), let *V*(*t*) > 0 at any moment of time *t* when system is active (otherwise, let *V*(*t*) = 0), and let the system evolution proceeds in time separated connected bursts of activity, called avalanches. Unlike in simulations, where the avalanches are clearly identified, the extraction of avalanches in experiments is obstructed due to a superimposed noise *n*(*t*) in measured signal *V*(*t*) + *n*(*t*). One way to overcome this obstacle is to impose some threshold, and subsequently analyze only the portion of signal above it. Compared to noise filtering as its alternative, the thresholding could be a preferred choice when the response signal is random.

**Figure 1:**
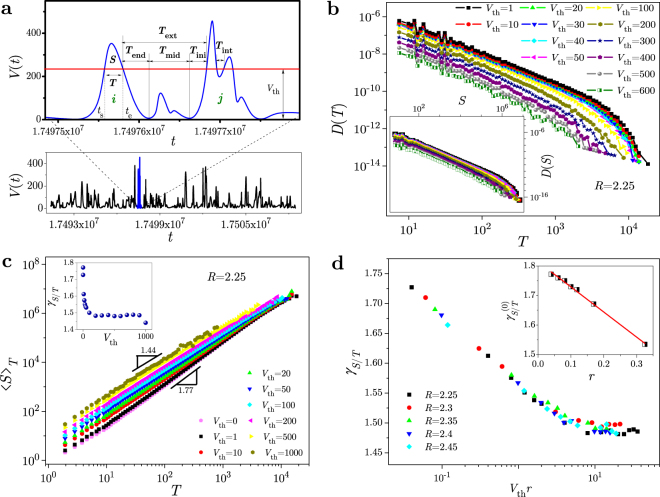
Thresholding of RFIM signal. The signal is obtained in simulations of 3D system with size *L* = 1024 and 40 random field configurations for each disorder *R*. (**a**) For a (blue) part of a train of avalanches (shown in bottom, and zoomed in top panel) and the imposed threshold *V*_th_ (red line), we illustrate: the determination of size *S* and duration *T* of a subavalanche (starting at the moment *t*_*s*_ and ending at *t*_*e*_ = *t*_*s*_ + *T*) taken out of the avalanche *i*, the internal waiting time *T*_int_ between two subavalanches of avalanche *j*, and the contributions *T*_end_, *T*_mid_, and *T*_ini_ to the external waiting time *T*_ext_ between avalanches *i* and *j*, see Eq. (1). (**b**) Distributions *D*(*T*) of duration (main panel), and distributions *D*(*S*) of size (inset) of subavalanches selected by thresholds from a wide range shown in legend. (**c**) 〈*S*〉_*T*_ shown against *T* for the thresholds in legend, where 〈*S*〉_*T*_ is the average size of subavalanches with duration *T*; variation of exponent *γ*_*S*/*T*_ with *V*_th_ is shown in inset. (**d**) *γ*_*S*/*T*_ vs *V*_th_ data, obtained for various disorders *R* (see legend), collapse onto a same curve when presented against *V*_th_*r*, where the reduced disorder *r* = (*R* − *R*_*c*_)/*R* measures a distance to the critical disorder *R*_*c*_ of the model. Inset shows how ${\gamma }_{S/T}^{(0)}$ (i.e. the exponent *γ*_*S*/*T*_ taken for *V*_th_ = 0) depends on the reduced disorder *r*.

When a threshold is imposed on a signal, the events of interest are connected bursts of activity above the threshold. Each such burst is a subavalanche of some underlying avalanche, comprising entire activity of the system at the current stage of its evolution. A subavalanche, selected by thresholding, begins at the moment of time when the signal exceeds the threshold, and ends when the signal falls below it. The difference between these two moments is taken as duration *T*, and the area between the imposed threshold and the portion of the signal above it as the size of subavalanche, $S={\int }_{0}^{T}\,dt[V({t}_{s}+t)-{V}_{{\rm{th}}}]$, see Fig. 1a, where *t* is the time measured from the start *t*_*s*_ of the subavalanche.

Once a threshold is applied, a portion of the signal will remain below it. This implies an introduction of the concept of waiting time *T*_*w*_, describing the time interval between two consecutive subavalanches, selected by the imposed threshold. Provided the start and end of each avalanche are known, like in simulations or in experiments after some minimal threshold is imposed, one can differentiate two kinds of waiting times. The internal waiting time is the waiting time *T*_int_ between two consecutive subavalanches thresholded from the same avalanche (avalanche *j* in Fig. 1a), while the external waiting time *T*_ext_ is the time between two consecutive subavalanches thresholded from two different avalanches (avalanches *i* and *j* in Fig. 1a).

A further distinction can be made among different types of contribution to the external waiting time *T*_ext_(*i*, *j*; *V*_th_); thus, see Fig. 1a,

1 $${T}_{{\rm{ext}}}(i,j;{V}_{{\rm{th}}})={T}_{{\rm{end}}}(i;{V}_{{\rm{th}}})+{T}_{{\rm{mid}}}(i,j;{V}_{{\rm{th}}})+{T}_{{\rm{ini}}}(j;{V}_{{\rm{th}}}),$$

where *T*_end_(*i*; *V*_th_) is the time taken by the avalanche *i* to end (i.e. fall from *V*_th_ to zero), *T*_mid_(*i*, *j*; *V*_th_) is the time spent by a whole sequence of consecutive avalanches that lie between the avalanches *i* and *j* and remain below *V*_th_ (note that this sequence may be empty), and *T*_ini_(*j*; *V*_th_) is the time taken by the avalanche *j* to rise from zero to *V*_th_.

In the main panel of Fig. 1b, we show the distributions *D*(*T*) of duration *T*, and in the inset - distributions *D*(*S*) of size *S* obtained in simulations of 3D RFIM systems with a single value of lattice size *L* = 1024 and disorder *R* = 2.25 which is above effective critical disorder ${R}_{c}^{{\rm{eff}}}\approx 2.21$ for the given system size (see Methods for more details). The distributions are collected for the subavalanches extracted above threshold *V*_th_ from the avalanches triggered in a zero-centered window of external magnetic field in which the response signal can be considered as stochastically stationary. We found that in a wide range of thresholds, both types of distribution follow power-laws $D(T)={T}^{-{\tau }_{T}}{g}_{T}(-T/{T}_{0})$ and $D(S)={S}^{-{\tau }_{S}}{g}_{S}(-S/{S}_{0})$, terminated by the cutoff scaling functions *g*_*T*_(*x*) and *g*_*S*_(*x*) for duration and size, respectively. The cutoff time *T*_0_, and the cutoff size *S*_0_, decrease when the threshold *V*_th_ increases. The values of exponents, pertaining to these distributions are: *τ*_*T*_ = 1.64 ± 0.02 for duration, and *τ*_*s*_ = 1.38 ± 0.03 for size of subavalanches. These values are obtained using ${g}_{T}(-T/{T}_{0})\propto \exp [-{(T/{T}_{0})}^{{\sigma }_{T}}]$, and ${g}_{S}(-S/{S}_{0})\propto \exp [-{(S/{S}_{0})}^{{\sigma }_{S}}]$, where *σ*_*T*_ and *σ*_*S*_ are the cutoff exponents whose values are close to 1 for all the distributions being analysed.

The average size 〈*S*〉_*T*_ of subavalanches with duration *T* is shown against *T* in the main panel of Fig. 1c for a family of curves, corresponding to different values of threshold *V*_th_. The graph demonstrates that ${\langle S\rangle }_{T}\sim {T}^{{\gamma }_{S/T}}$ in a broad range of thresholds, and that the power-law exponent *γ*_*S*/*T*_ varies with threshold. As can be seen in inset, when the threshold increases, the exponent *γ*_*S*/*T*_ decreases (from the value 1.77 for very low thresholds, to the value of 1.44 for very high threshold levels), forming some sort of plateau, like in the crack-line propagation model^30^.

In order to gain a more complete insight into the variation of *γ*_*S*/*T*_ with *V*_th_, we show in the main panel of Fig. 1d its values against *V*_th_*r*, i.e. threshold multiplied by *r*, where *r* is the reduced disorder *r* ≡ (*R* − *R*_*c*_)/*R*, measuring the distance to the critical disorder *R*_*c*_ of the model. The data obtained for different disorders collapse onto a same curve, suggesting that a joint plateau is formed at the value ${\gamma }_{S/T}^{({\rm{pl}})}=1.49\pm 0.02$. The existence of plateau may be considered as an important feature of the model, because the plateau value ${\gamma }_{S/T}^{({\rm{pl}})}$ remains stable under variation of both threshold and disorder unlike, for instance, the value ${\gamma }_{S/T}^{({\rm{0}})}$ of exponent *γ*_*S*/*T*_ for zero threshold, which (seeming linearly) changes with disorder - see the inset of Fig. 1d. Having at hand two well defined values, ${\gamma }_{S/T}^{({\rm{0}})}$ and ${\gamma }_{S/T}^{({\rm{pl}})}$, one could interpret the variation of *γ*_*S*/*T*_-values, presented in Fig. 1c, as a possible crossover between these two characteristic values.

**Figure 2:**
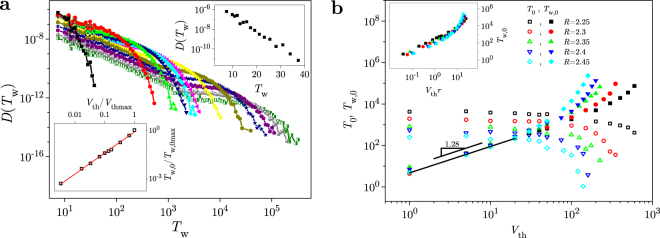
Threshold induced correlations in waiting times distributions. In panel (**a**) we show the distributions *D*(*T*_w_) of the waiting time *T*_w_ for a wide range of threshold levels, and for the same set of data as in Fig. 1. The cutoff waiting time *T*_w,0_ of distributions *D*(*T*_w_) grows with *V*_th_ as ${T}_{{\rm{w}},{\rm{0}}}\propto {V}_{{\rm{th}}}^{\delta }$, where *δ* = 1/(*γ*_*S*/*T*_ − 1) and *δ* = 1.30 ± 0.02 (see the bottom inset). As indicated in the top inset, for the case of the lowest threshold, *D*(*T*_w_) is approximately exponential, while upon increasing the *V*_th_, a power law form gradually develops, as can be seen in the main panel. In panel (**b**) we give the cutoff time and the cutoff waiting time for different values of disorder *R* (all above the effective critical disorder), and in inset the scaling collapse of the *T*_w,0_ vs *V*_th_ curves, pertaining to different distances *r* to the critical disorder.

The distributions *D*(*T*_w_) of waiting time *T*_w_, presented in panel **a** of Fig. 2, are obtained from the same data and for the same thresholds as for the distributions shown in Fig. 1b. They (also) follow power-laws $D({T}_{{\rm{w}}})={T}^{-{\tau }_{{T}_{{\rm{w}}}}}{g}_{w}({T}_{{\rm{w}}}/{T}_{{\rm{w}},0})$, specified by the value ${\tau }_{{T}_{{\rm{w}}}}=1.64\pm 0.02$ for the exponent of the waiting time, and terminated by the cutoff scaling function *g*_*w*_(*x*), taken for *T*_w_/*T*_w,0_, where *T*_w,0_ is the cutoff waiting time. In contrast to the cutoff time *T*_0_ which decreases with threshold, the cutoff waiting time *T*_w,0_ increases with *V*_th_. This is shown in the bottom inset of Fig. 2a, where one can see that the relation ${T}_{{\rm{w}}\mathrm{,0}}\sim {V}_{{\rm{th}}}^{\delta }$ is satisfied with *δ* ≈ 1.30 ± 0.02.

Regarding the shape of distributions *D*(*T*_w_), one can see that the power law part appears with the increase of threshold, indicating the onset of correlations due to avalanches that are partially hidden below the detection threshold. Opposite to that, one can notice that the power law gradually vanishes for the very low threshold levels due to very small value of the cutoff waiting time *T*_w,0_. This is illustrated in the top inset of Fig. 2a, where the power law reduces to the (approximately) exponential cutoff scaling function *g*_*w*_(*T*_w_/*T*_w,0_). The distribution of waiting times *D*(*T*_w_) for the zero threshold, should be given by a delta function limit of the exponential distribution with vanishing cutoff waiting time *T*_w,0_, due to specific pattern of driving explained in Methods, giving no time separation between consequtive avalanches.

Finally, in Fig. 2 panel **b**, we present the cutoff time *T*_0_ and the cutoff waiting time *T*_w,0_ against threshold *V*_th_ for a family of curves obtained for different disorders *R*. For small thresholds, the cutoff waiting time grows as a power law (${T}_{{\rm{w}},0}\sim {V}_{{\rm{th}}}^{1.3}$), and more rapidly than that for larger *V*_th_. This leads to the conclusion that if the threshold is so high that only the avalanches from the cutoff of the “true” distribution are “observable”, then a very large separation between the time scales of the waiting times and avalanche durations may ensue^32^. Pertaining scaling collapse of the cutoff waiting times, corresponding to different disorders, is obtained by multiplying the threshold axis by *r*, as is shown in the inset.

### Scaling theory of avalanche correlations

Scaling properties of the threshold induced correlations can be predicted for signals emitted by a wide range of systems that respond to stationary external driving in stochastically stationary trains of avalanches. To this end, let *V*(*t*) be a response signal that continuously varies with (continuous) time *t*, and let *V*(*t*) > 0 at any moment of time when the system is active, while *V*(*t*) = 0 otherwise. Such signal is a sequence of avalanches, separated by intervals of time when the system is quiet, each avalanche being a continuous burst of values *V*(*t*) > 0 taken at any *t*_*s*_ < *t* < *t*_*e*_ between the moments *t*_*s*_ and *t*_*e*_ when the avalanche starts/ends, and therefore *V*(*t*_*s*_) = *V*(*t*_*e*_) = 0. Essentially the same can be said for discrete signals sampled at discrete moments of time, provided that they are continuously (e.g. linearly) extrapolated to all moments of time throughout the signal duration, like it is done here with the RFIM signals.

The avalanches can be classified into types so that all avalanches of the same type have the same profile *f*(*t*′) ≡ *V*(*t*_*s*_ + *t*′) with respect to time *t*′ measured from their start. Using this equivalence relation, one can obtain the set of all avalanche types $\Im$, and introduce a one-parameter family of scaling transformations ${\hat{{\rm{S}}}}_{b}:\Im \to \Im$, specified by the real-valued parameter *b* > 0, such that the time profile ${f}_{{\hat{{\rm{S}}}}_{b}i}(t^{\prime} )$ of the scaled avalanche type ${\hat{{\rm{S}}}}_{b}i$ is

2 $${f}_{{\hat{{\rm{S}}}}_{b}i}(t^{\prime} )\equiv {b}^{x}{f}_{i}(bt^{\prime} ),$$

where *x* is an exponent specified by the type of the involved system^5^. For this family of transformations one can prove that

3 $${T}_{{\hat{{\rm{S}}}}_{b}i}={T}_{i}/b,\quad {S}_{{\hat{{\rm{S}}}}_{b}i}={b}^{x-1}{S}_{i},$$

where *T*_*i*_ is duration, and ${S}_{i}\equiv {\int }_{0}^{{T}_{i}}\,{f}_{i}(t^{\prime} )dt^{\prime}$ is the size of the avalanche type *i*, while ${T}_{{\hat{{\rm{S}}}}_{b}i}$ and ${S}_{{\hat{{\rm{S}}}}_{b}i}$ are duration and size of the scaled avalanche type ${\hat{{\rm{S}}}}_{b}i$, respectively. Analogous expressions

4 $$T({\hat{{\rm{S}}}}_{b}i;{b}^{x}{V}_{{\rm{th}}})=T(i;{V}_{{\rm{th}}})/b,\quad S({\hat{{\rm{S}}}}_{b}i;{b}^{x}{V}_{{\rm{th}}})={b}^{x-1}S(i;{V}_{{\rm{th}}}),$$

hold for the duration *T*(*i*; *V*_th_) and size $S(i;{V}_{{\rm{th}}})={\int }_{{t}_{s}}^{{t}_{e}}\,{f}_{i}(t^{\prime} )dt^{\prime} -{V}_{{\rm{th}}}T(i;{V}_{{\rm{th}}})$, and also for the following types of waiting times:

5 $$\begin{array}{rcl}{T}_{{\rm{int}}}({\hat{{\rm{S}}}}_{b}i;{b}^{x}{V}_{{\rm{th}}}) & = & {T}_{{\rm{int}}}(i;{V}_{{\rm{th}}})/b,\\ {T}_{{\rm{ini}}}({\hat{{\rm{S}}}}_{b}i;{b}^{x}{V}_{{\rm{th}}}) & = & {T}_{{\rm{ini}}}(i;{V}_{{\rm{th}}})/b,\\ {T}_{{\rm{end}}}({\hat{{\rm{S}}}}_{b}i;{b}^{x}{V}_{{\rm{th}}}) & = & {T}_{{\rm{end}}}(i;{V}_{{\rm{th}}})/b,\end{array}$$

all for the subavalanches extracted above the threshold *V*_th_ out of avalanche of type *i*, and for the corresponding quantities $T({\hat{{\rm{S}}}}_{b}i;{b}^{x}{V}_{{\rm{th}}})$, $S({\hat{{\rm{S}}}}_{b}i;{b}^{x}{V}_{{\rm{th}}})$, ${T}_{{\rm{int}}}({\hat{{\rm{S}}}}_{b}i;{b}^{x}{V}_{{\rm{th}}})$, ${T}_{{\rm{ini}}}({\hat{{\rm{S}}}}_{b}i;{b}^{x}{V}_{{\rm{th}}})$, and ${T}_{{\rm{end}}}({\hat{{\rm{S}}}}_{b}i;{b}^{x}{V}_{{\rm{th}}})$ describing the scaled avalanche ${\hat{{\rm{S}}}}_{b}i$, extracted above the scaled threshold ${b}^{x}{V}_{{\rm{th}}}$. Furthermore, if one scales the whole portion of response signal, starting with an avalanche (of type *i*) that surpasses threshold *V*_th_, and ending with successive avalanche (of type *j*) above the same threshold, then analogous expression hold for the waiting time *T*_mid_(*i*, *j*; *V*_th_), spent by the avalanches that lie between these avalanches and remain below *V*_th_, namely:

6 $${T}_{{\rm{mid}}}({\hat{{\rm{S}}}}_{b}i,{\hat{{\rm{S}}}}_{b}j;{b}^{x}{V}_{{\rm{th}}})={T}_{{\rm{mid}}}(i,j;{V}_{{\rm{th}}})/b,$$

which, combined with Eqs (1) and (5), gives the same type of scaling

7 $${T}_{{\rm{ext}}}({\hat{{\rm{S}}}}_{b}i,{\hat{{\rm{S}}}}_{b}i;{b}^{x}{V}_{{\rm{th}}})={T}_{{\rm{ext}}}(i,j;{V}_{{\rm{th}}})/b,$$

for the external waiting time.

For avalanches of each type, one can also count the average number ${n}_{i}^{(T)}(T;{V}_{{\rm{th}}})$ in the response signals of their subavalanches that are above the threshold *V*_th_, and have the duration which is not greater than *T*. Likewise, one can further find the numbers ${n}_{i}^{(S)}(S;{V}_{{\rm{th}}})$ of subavalanches with size not greater than *S*, and the numbers ${n}_{i}^{({T}_{{\rm{int}}})}({T}_{{\rm{int}}};{V}_{{\rm{th}}})$, ${n}_{i}^{({T}_{{\rm{end}}})}({T}_{{\rm{end}}};{V}_{{\rm{th}}})$, and ${n}_{i}^{({T}_{{\rm{ini}}})}({T}_{{\rm{ini}}};{V}_{{\rm{th}}})$ corresponding to internal waiting time *T*_int_, and contributions *T*_end_ and *T*_ini_ to the external waiting time, respectively. These numbers obey:

8 $${n}_{{\hat{{\rm{S}}}}_{b}i}^{(T)}(T/b;{b}^{x}{V}_{{\rm{th}}})={n}_{i}^{(T)}(T;{V}_{{\rm{th}}}),\quad {n}_{{\hat{{\rm{S}}}}_{b}i}^{(S)}({b}^{x-1}S;{b}^{x}{V}_{{\rm{th}}})={n}_{i}^{(S)}(S;{V}_{{\rm{th}}}),$$

9 $${n}_{{\hat{{\rm{S}}}}_{b}i}^{({T}_{{\rm{int}}})}({T}_{{\rm{int}}}/b;{b}^{x}{V}_{{\rm{th}}})={n}_{i}^{({T}_{{\rm{int}}})}({T}_{{\rm{int}}};{V}_{{\rm{th}}}),$$

10 $${n}_{{\hat{{\rm{S}}}}_{b}i}^{({T}_{{\rm{end}}})}({T}_{{\rm{end}}}/b;{b}^{x}{V}_{{\rm{th}}})={n}_{i}^{({T}_{{\rm{end}}})}({T}_{{\rm{end}}};{V}_{{\rm{th}}}),\quad {n}_{{\hat{{\rm{S}}}}_{b}i}^{({T}_{{\rm{ini}}})}({T}_{{\rm{ini}}}/b;{b}^{x}{V}_{{\rm{th}}})={n}_{i}^{({T}_{{\rm{ini}}})}({T}_{{\rm{ini}}};{V}_{{\rm{th}}}).$$

Next, let *dp*(*i*; *λ*) be an elementary probability of obtaining an avalanche of type *i* in the response signal under observation conditions specified by some appropriate multiparameter *λ* = (*λ*_1_, *λ*_2_, …*λ*_*n*_), like *λ* = (*h*′, *r*, 1/*L*) for the RFIM signals (*L* is system size, *r* is the reduced disorder, and *h*′ is the reduced magnetic field, see Methods). Having at our disposal this probability, one can express the distribution *D*_*T*_(*T*; *V*_th_, *λ*) of subavalanches that are obtained under conditions *λ* and have the duration *T* above the threshold *V*_th_. Thus,

11 $${D}_{T}(T;{V}_{{\rm{th}}},\lambda )={C}_{T}\,\int \,\frac{d{n}_{i}^{(T)}(T;{V}_{{\rm{th}}})}{dT}dp(i;\lambda ),$$

where *C*_*T*_ is an appropriate normalization constant, and analogously for other similar distributions *D*_*S*_(*S*; *V*_th_, *λ*) and ${D}_{{T}_{{\rm{int}}}}({T}_{{\rm{int}}};{V}_{{\rm{th}}},\lambda )$ for size *S* and internal waiting time *T*_int_, as well as for the distributions ${D}_{{T}_{{\rm{end}}}}({T}_{{\rm{end}}};{V}_{{\rm{th}}},\lambda )$ and ${D}_{{T}_{{\rm{ini}}}}({T}_{{\rm{ini}}};{V}_{{\rm{th}}},\lambda )$ of *T*_end_ and *T*_ini_.

Now, let us suppose that the elementary probability $dp({\hat{{\rm{S}}}}_{b}i;{b}^{\zeta }\lambda )$ of obtaining the scaled type ${\hat{{\rm{S}}}}_{b}i$ satisfies

12 $$dp({\hat{{\rm{S}}}}_{b}i;{b}^{\zeta }\lambda )={b}^{w}dp(i;\lambda ),$$

where *w* is a probability exponent, and *ζ* = (*ζ*_1_, *ζ*_2_, …, *ζ*_*n*_) is a multiexponent, so that ${b}^{\zeta }\lambda \equiv ({b}^{{\zeta }_{1}}{\lambda }_{1},{b}^{{\zeta }_{2}}{\lambda }_{2},\ldots ,{b}^{{\zeta }_{n}}{\lambda }_{n})$ are the scaled conditions under which the type ${\hat{{\rm{S}}}}_{b}i$ is observed. Starting from this expression, which is in fact a generalized scaling hypothesis, one can obtain the scaling laws

13 $${D}_{T}(T/b;{b}^{x}{V}_{{\rm{th}}},{b}^{\zeta }\lambda )={b}^{w+1}{D}_{T}(T;{V}_{{\rm{th}}},\lambda ),\quad {D}_{S}({b}^{x-1}S;{b}^{x}{V}_{{\rm{th}}},{b}^{\zeta }\lambda )={b}^{w+1-x}{D}_{S}(S;{V}_{{\rm{th}}},\lambda ),$$

14 $${D}_{{T}_{{\rm{int}}}}({T}_{{\rm{int}}}/b;{b}^{x}{V}_{{\rm{th}}},{b}^{\zeta }\lambda )={b}^{w+1}{D}_{{T}_{{\rm{int}}}}({T}_{{\rm{int}}};{V}_{{\rm{th}}},\lambda ),$$

15 $$\begin{array}{rcl}{D}_{{T}_{{\rm{end}}}}({T}_{{\rm{end}}}/b;{b}^{x}{V}_{{\rm{th}}},{b}^{\zeta }\lambda ) & = & {b}^{w+1}{D}_{{T}_{{\rm{end}}}}({T}_{{\rm{end}}};{V}_{{\rm{th}}},\lambda ),\\ {D}_{{T}_{{\rm{ini}}}}({T}_{{\rm{ini}}}/b;{b}^{x}{V}_{{\rm{th}}},{b}^{\zeta }\lambda ) & = & {b}^{w+1}{D}_{{T}_{{\rm{ini}}}}({T}_{{\rm{ini}}};{V}_{{\rm{th}}},\lambda \mathrm{).}\end{array}$$

The foregoing general predictions can be tested in the case of any response signal for which can be expected that the assumptions, used in their derivation, are reasonably satisfied. The first step towards that in the case of RFIM signal is to specify the observation conditions *λ*, and next to express the generic exponents *x*, *y*, *w*, and *ζ* in the terms of standard RFIM exponents. Here, as we already mentioned, the observation conditions are *λ* = (*h*′, *r*, 1/*L*), while for the exponents *x* and *w*, and for the multiexponent *ζ* = (*ζ*_*h*_, *ζ*_*r*_, *ζ*_*L*_), we found that

16 $$x=1-\mathrm{1/}\sigma \nu z,\quad w=\alpha -1,$$

17 $${\zeta }_{h}=\beta \delta /\nu z,\quad {\zeta }_{r}=\mathrm{1/}\nu z,\quad {\zeta }_{L}=\mathrm{1/}z,$$

where *σ*, *ν*, *z*, *α*, *β* and *δ* are the standard RFIM exponents^3,9,10^.

**Figure 3:**
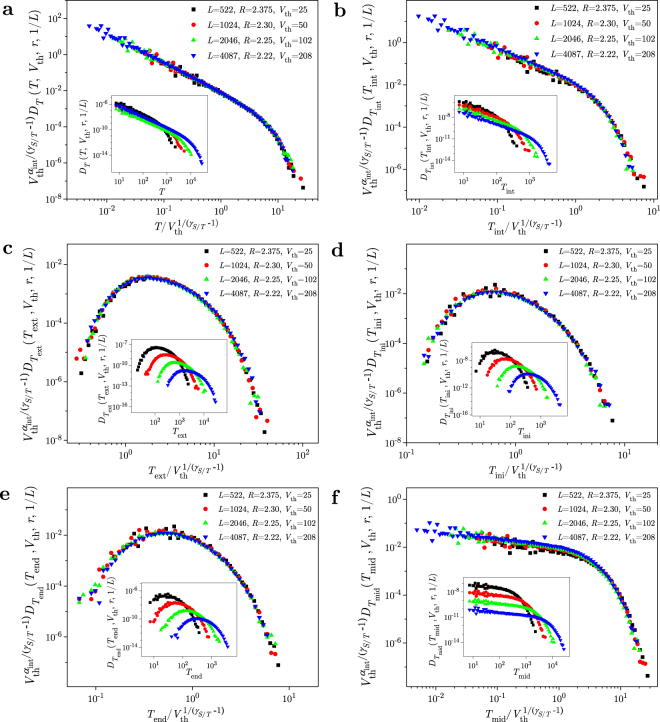
Collapse of duration and waiting time distributions. The distributions correspond to subavalanches that are above threshold *V*_th_, and are collected in RFIM simulations of 3D systems with lattice sizes *L* and disorders $R > {R}_{c}^{{\rm{eff}}}$, given in legends and satisfying collapsing requirements Eq. (19). All collapses are achieved after the distributions are multiplied by ${V}_{{\rm{th}}}^{{\alpha }_{{\rm{int}}}/({\gamma }_{S/T}-\mathrm{1)}}$ and presented against their arguments divided by ${V}_{{\rm{th}}}^{\mathrm{1/(}{\gamma }_{S/T}-\mathrm{1)}}$, see Eqs (18) and (20). In panel (**a**) we show the scaling collapse of distributions of subavalanche durations, and in panels (b–f) collapsing of distributions corresponding to the following types of waiting times: *T*_int_, *T*_ext_, *T*_ini_, *T*_end_, and *T*_mid_, respectively. Original distributions are shown in insets.

In Fig. 3 we present the collapsing for distributions of duration and of various types of waiting times, all for the subavalanches above thresholds. The subavalanches are taken from a family of response signals observed under conditions which are aligned according to the collapsing requirements together with the corresponding collapsing predictions. Thus, in panel a, the data are scaled in agreement with

18 $${V}_{{\rm{th}}}^{{\alpha }_{{\rm{int}}}/({\gamma }_{S/T}-\mathrm{1)}}{D}_{T}(T;{V}_{{\rm{th}}},r,1/L)={D}_{T}(T/{V}_{{\rm{th}}}^{\mathrm{1/(}{\gamma }_{S/T}-\mathrm{1)}};{V}_{{\rm{th}}}^{\sigma \mathrm{/(1}-{\gamma }_{T/S})}r,{V}_{{\rm{th}}}^{\sigma \nu \mathrm{/(1}-{\gamma }_{T/S})}/L),$$

which predicts that the distribution data, multiplied by ${V}_{{\rm{th}}}^{{\alpha }_{{\rm{int}}}/({\gamma }_{S/T}-\mathrm{1)}}$ and presented against $T/{V}_{{\rm{th}}}^{\mathrm{1/(}{\gamma }_{S/T}-\mathrm{1)}}$, fall onto a same curve, if the distributions are obtained for disorders *R* and lattice sizes *L*, satisfying the collapsing requirements:

19 $${V}_{{\rm{th}}}^{\sigma \mathrm{/(1}-{\gamma }_{T/S})}r=const,\quad {V}_{{\rm{th}}}^{\sigma \nu \mathrm{/(1}-{\gamma }_{T/S})}/L=const\mathrm{.}$$

Here, *α*_int_ = *α* + *σβδ*/*γ*_*T*/*S*_, and ${\gamma }_{T/S}=\mathrm{1/}{\gamma }_{S/T}=\sigma \nu z$, are the standard RFIM exponents^3,9^, and *α*_int_ is used because the data are collected in the windows having finite width of external magnetic field^10^. The same form of data collapsing is also followed by the distributions of all types of waiting times, namely

20 $${V}_{{\rm{th}}}^{{\alpha }_{{\rm{int}}}/({\gamma }_{S/T}-\mathrm{1)}}{D}_{{T}_{{\rm{w}}}}({T}_{{\rm{w}}};{V}_{{\rm{th}}},r,1/L)={D}_{{T}_{{\rm{w}}}}({T}_{{\rm{w}}}/{V}_{{\rm{th}}}^{\mathrm{1/(}{\gamma }_{S/T}-\mathrm{1)}};{V}_{{\rm{th}}}^{\sigma \mathrm{/(1}-{\gamma }_{T/S})}r,{V}_{{\rm{th}}}^{\sigma \nu \mathrm{/(1}-{\gamma }_{T/S})}/L),$$

where *T*_w_ stands for *T*_int_, *T*_ext_, *T*_ini_ or *T*_end_, and this is presented in the remaining panels b–e of Fig. 3. Here, we would like to remark that the generalized scaling hypothesis Eq. (12) is not sufficient to predict the scaling for the distribution of *T*_mid_ waiting time. Nevetheless, our data indicate that the same type of scaling also holds for this distribution, as is shown in panel **f** of Fig. 3, which might indicate that Eq. (12) holds not only for individual avalanche types, but also for larger portions of the response signal.

## Discussion

Our results demonstrate that when a thresholding procedure is applied to a RFIM signal at T = 0, temporal correlations emerge between the avalanches. These correlations are detectable in a form of power-law distributed waiting times and are a result of original avalanches being separated into subavalanches due to the thresholding. These power-law parts of waiting time distributions gradually vanish as the value of the threshold decreases, down to the lowest threshold when the distribution of waiting times turns approximately exponential. This implies that even though the avalanches are triggered by a random process, applying a finite threshold implicitly introduces underlying temporal correlations in a given signal. On the other hand, distributions of the subavalanche durations and sizes follow power law with cutoffs decreasing when the threshold level increases. Thus the present paper verifies the fact that the scaling predictions obtained for the previously studied crack-line propagation model^30,32^ also hold in the case of RFIM signals, suggesting their general validity in accordance with the scaling hypothesis and derived scaling forms proposed in the previous section.

In our analysis we have also identified different contributions of waiting times (*T*_int_, *T*_ext_, *T*_ini_, *T*_end_, *T*_mid_), which all follow the same scaling form Eq. (20). This form predicts that rescaled distributions of each type of waiting time, obtained for system parameters adjusted according to the collapsing requirements, all collapse onto a single curve. As the proposed general scaling theory predicts, we anticipate that the derived scaling forms of the threshold induced correlations, provided that the requirements of the theory are fulfilled, should hold for any response signal originating from system that, as a response to slowly changing external conditions, relaxes in an avalanche-like intermittent way. For such response signals, one can say that, in general, the external waiting times are affected by the two mechanisms: (i) implicit or explicit application of a finite threshold resulting in low levels of avalanche activity not being detected, and (ii) effects due to details of the implementation of the external driving. While we focus here on the first mechanism, a more detailed study of the interplay between the two, considering the joint effects of finite thresholds and driving rates, would be an interesting avenue of future work.

Additionally, we have found that the exponent *γ*_*S*/*T*_, obtained from the scaling of the average avalanche size with duration, is affected by the level of the applied theshold, with theoretically expected value of 1.77 recovered only in the limit of very low thresholds. The effective value of *γ*_*S*/*T*_ deviates from this value in a way that it initially decreases and then reaches the plateau where it remains stable for a wide range of threshold and disorder values. This result is of special importance for analysis of experimental results where setting a finite detection threshold is an inevitable procedure in order to be able to perform the analysis of the recorded signal.

Qualitatively speaking, our results meet very well to the ones obtained for the formerly studied crack-line propagation model^30,32^. Given that our numerically generated data are noise-free, we anticipate the possibility to observe in real experiments the additional effects that the presence of noise could impose on the thresholded signal^28^. Thus, our work calls for further and more detailed investigation of experimental data in order to unravel the true nature of underlaying mechanisms causing the onset of temporal correlations in these systems.

## Methods

### Simulations of the Random Field Ising Model

In order to investigate the threshold induced temporal correlations in the Random Field Ising Model (RFIM) we performed numerical simulations of its athermal (*T* = 0) variant in nonequilibrium adiabatic regime. The athermal RFIM describes a system of *N* ferromagnetically coupled classical Ising spins *S*_*i*_ = ±1, located at sites *i* of some underlying lattice. The spins are influenced by a homogeneous external magnetic field *H*, and by some local magnetic field *h*, whose values *h*_*i*_ vary randomly from site to site due to random distribution of quenched impurities that generate that field. Therefore, it is considered that each instance of RFIM system is specified by the configuration of values ${\{{h}_{i}\}}_{i=1}^{N}$ that the quenched random field *h* takes at the sites *i* of that system, and that these values remain frozen throughout any evolution of the system.

In the basic RFIM case, presented here, the ferromagnetic coupling between spins extends only to the nearest-neighbors, so the effective magnetic field acting on spin *S*_*i*_ is

21 $${h}_{i}^{{\rm{eff}}}=J\,\sum _{j}\,{S}_{j}+H+{h}_{i},$$

where *S*_*j*_ are the nearest neighbors of *S*_*i*_, and *J* > 0 is a ferromagnetic coupling constant. Hence, the system Hamiltonian reads

22 $$ {\mathcal H} =-J\,\sum _{\langle i,j\rangle }\,{S}_{i}{S}_{j}-H\,\sum _{i}\,{S}_{i}-\sum _{i}\,{h}_{i}{S}_{i},$$

and in this expression the first sum refers only to the nearest-neighbor spins *S*_*i*_ and *S*_*j*_, the second term describes the coupling of spins with the external field *H*, while the last term gives the coupling with quenched random field *h*. At any site *i*, its value *h*_*i*_ is taken randomly from some zero-centered distribution, same for all sites, and for any two different sites *i* ≠ *j* the values *h*_*i*_ and *h*_*j*_ are chosen independently, so that the expected value of their product is 〈*h*_*i*_*h*_*j*_〉 = 0. For generating the values of the quenched random field, here we use a Gaussian distribution

23 $$\rho (h)=\frac{1}{\sqrt{2\pi }R}\,\exp \,(-\frac{{h}^{2}}{2{R}^{2}}),$$

and take its standard deviation $R=\sqrt{\langle {h}^{2}\rangle }$ as a measure of disorder in the system.

In the nonequilibrium athermal RFIM, the system evolves according to the following flipping rule: each spin *S*_*i*_ remains stable while its sign equals the sign of the effective field ${h}_{i}^{{\rm{eff}}}$ at its site; otherwise, *S*_*i*_ becomes unstable, and flips at the next moment *t* + 1 of discrete time. Thus, the flipping of each spin influences the effective field for all of its nearest neighbors. All those neighbors that become unstable will flip at the next moment of time, which in turn may cause the flipping of their neighbors, making an avalanche which lasts until all spins become stable.

Once all spins become stable, the only way to trigger a new avalanche is to change the external field *H*, and in this way drive the system by a sequence of *H*-increments, forming a driving pattern that is set in advance. Typically, the changes between two consecutive moments of time are small, resembling the usual real-world situation with two well separated time scales: fast one for spin flipping, and slow scale for the external field. In the limiting regime of infinitely slow (i.e. adiabatic) driving, the external field is kept constant during (any) avalanche. After the avalanche dies, *H* continues to change (i.e. increase or decrease following the current direction of driving pattern) until it reaches exactly the value that triggers only the least stable spin. Note, however, that because all spins during the foregoing change remain stable, and therefore unaltered, the overall change of *H* is allowed to be done in a single jump, which is utilized in computer simulations for better efficiency. The consequence of such driving pattern is that the next avalanche is triggered immediately after the previous one has ended.

Together with the driving pattern, one also needs to specify some initial and final conditions. Here, we take that initially *H* = −∞ and all spins are −1, and then we gradually increase *H* until all spins become +1. At each moment of time *t*, we register the number of spins *V*(*t*) flipped at that moment, and in this way collect system’s response along the whole rising part of the saturation hysteresis loop. Note that if one repeats the run with the same sample (i.e. same configuration ${\{{h}_{i}\}}_{i=1}^{N}$ of random field), in the same driving regime, and with the same initial conditions, the system response will be the same because the flipping rule is deterministic. Therefore, reliable avalanche statistics are collected by repeating the whole procedure many times using different random field configurations (quenched or sample averaging).

Our RFIM simulations are done in the nonequilibrium adiabatic regime with *J* = 1, and with closed boundary conditions on 3D lattices *L* × *L* × *L* of linear size *L* = 1024. For the analysis of the effects of the of imposed threshold *V*_th_, we have used the parts of signal where the signal can be considered to be approximately stationary. This is fulfilled in the narrow window of external field *H*, taken around the coercive value (i.e. the value at which the system magnetization $M={\sum }_{i=1}^{N}\,{S}_{i}$ is zero). One fragment of such signal is shown in the bottom panel of Fig. 1a. The number of spins flipped at a given moment of time *t* is taken as the signal value *V*(*t*) at that moment, and the time is measured from the window start. The statistics, collected by quenched averaging for given disorder *R*, are described using the scaling variables: reduced disorder *r* = (*R* − *R*_c_)/*R* and reduced magnetic field *h* = *H* − *H*_c_ − *b*_r_*r*, where *R*_c_ is the critical disorder, *H*_c_ is the critical value of magnetic field *H*, and *b*_r_ is the rotational parameter accounting how the effective critical value ${H}_{c}^{{\rm{eff}}}(r)$ of the external field (i.e. the value of *H* at which the maximum of susceptibility occurs), shifts with reduced disorder *r*^3,10^. In this paper we have confined our study to disorders that are above the effective critical disorder ${R}_{c}^{{\rm{eff}}}$ pertaining to the underlying lattices^33,34^. For this value of disorder, precisely defined in the quoted references, it is likely for the spanning avalanches to occur. The spanning avalanche is the avalanche that spans the finite system along at least one of its dimensions and therefore plays the role of infinite avalanche, causing the jump of magnetization in infinite systems below the critical disorder. In three-dimensional RFIM systems, these avalanches have different distributions than remaining (i.e. non-spanning) avalanches^35^, and violate the scaling assumptions given in section Scaling theory of avalanche correlations.

### Data availability

The datasets generated during and analysed during the current study are available from the corresponding author on reasonable request.
